# The Mechanism of MAPK Signal Transduction Pathway Involved with Electroacupuncture Treatment for Different Diseases

**DOI:** 10.1155/2019/8138017

**Published:** 2019-07-31

**Authors:** Weibin Du, Huahui Hu, Jiangsong Zhang, Guanai Bao, Rongliang Chen, Renfu Quan

**Affiliations:** ^1^Research Institute of Orthopedics, The Affiliated Jiangnan Hospital of Zhejiang Chinese Medical University, Hangzhou 312001, China; ^2^Research Institute of Acupuncture, Zhejiang Chinese Medical University, Hangzhou 310058, China; ^3^Zhejiang Cancer Hospital, Hangzhou 310022, China

## Abstract

The mitogen-activated protein kinase (MAPK) signal transduction pathway plays an important role in the regulation of various diseases, such as cardiovascular and cerebrovascular diseases, and takes part in anti-inflammatory effects, analgesic effects, protection against injury, and maintenance of gastrointestinal functions. Electroacupuncture therapy is an external therapy used in traditional Chinese medicine. By adding external electrical stimulation to traditional acupuncture, the stimulus gets doubled and the therapeutic efficacy gets enhanced accordingly. It combines the benefits of both acupuncture and electrical stimulation. In recent years, some studies have explored the molecular mechanisms of MAPK signal pathways involved with electroacupuncture treatment. Based on these recent studies, this article summarizes the mechanisms of MAPK signal transduction pathways involved with electroacupuncture treatment. This adds great value to the studies of molecular mechanisms of electroacupuncture treatment and also provides an effective reference for its clinical use.

## 1. Introduction

**Figure 1:**
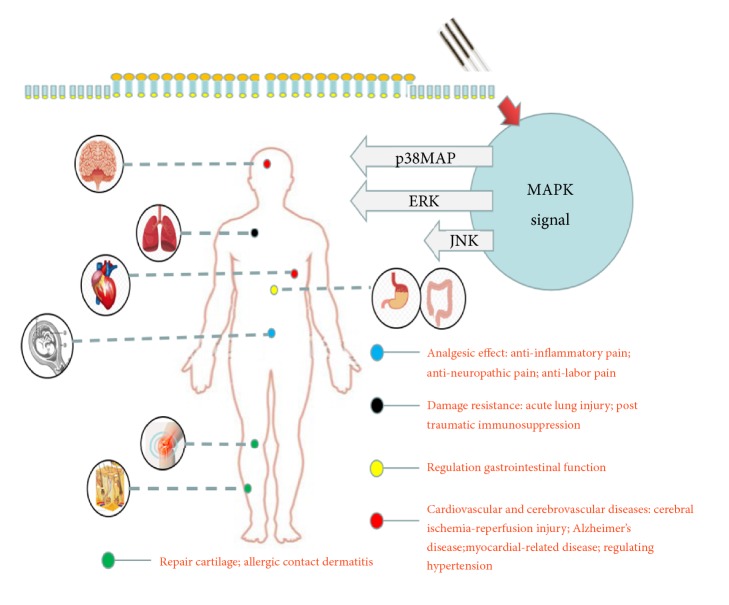
MAPK signal transduction pathways: key factors in acupuncture treatment for related diseases.

Electroacupuncture is an emerging therapy that combines acupuncture with electrical stimulation. By varying factors including electroacupuncture wave, frequency, and stimulation time, scholars have deepened their understanding of the mechanism of disease treatment up to the levels of cells, molecules, proteins, and genes. At present, the correlation between electroacupuncture regulation and MAPK-related signaling pathways is mainly focused on the anti-inflammatory and analgesic effects, protection against injury, regulation of gastrointestinal functions, and prevention and treatment of cardiovascular and cerebrovascular diseases, skin diseases, and knee joint diseases. However, most of these researches have focused on experimental animals, and there are few clinical reports regarding this. Therefore, we reviewed the current relevant literature and analyzed the efficacy and mechanisms of electroacupuncture therapy related to the MAPK signal transduction pathway to help in the clinical treatment (Figure 1 and Table 1).

## 2. The Mechanism of MAPK Signal Transduction Pathway

The MAPK pathway is an important signaling system that mediates cellular responses. It is ubiquitous in various organisms including yeast and mammalian cells and is involved in many physiological processes, such as cell growth, development, division, and death and synchronization of cellular functions [1, 2]. Mitogen-activated protein kinase (MAPK) belongs to the family of silk proteins/threonine kinases. It is located in the cytoplasm and can enter the nucleus and once enabled can activate the target organ. MAPK-related signaling pathway has been confirmed to play an important role in cell proliferation, differentiation, and induction of apoptosis [3, 4]. The three subpathways of MAPK including p38 mitogen-activated protein kinase (p38MAPK), extracellular signal-regulated kinase (ERK-1/2), and c-Jun-terminal kinase (JNK), are most widely studied among the many subfamilies of the MAPK family [5, 6]. The p38MAPK and JNK signaling pathways are mainly activated under stress and play roles in growth inhibition, inflammation, and proapoptotic signaling. In contrast, the ERK signaling pathway mainly exerts its effects on cell proliferation, development, and differentiation induction [7, 8].

## 3. Analgesic Effects of Electroacupuncture Based on MAPK Signal Transduction Pathway 

### 3.1. Anti-Inflammatory and Analgesic Effects of Electroacupuncture

The pain mediated by nociceptors caused by the continuous stimulus is mainly inflammatory in nature. Previous studies have shown that electroacupuncture treatment in inflammatory pain can be explained by two mechanisms of action: peripheral (endogenous opioid peptide, COX-2, serotonin, nerve growth factor, and bradykinin) and centric (spinal cord-related pathway, and central depression inhibitory system). At present, studies [9–12] on the mechanism of analgesic actions of acupuncture from the angle of MAPK signal transduction mostly involve the classic complete Freund's adjuvant (CFA) rats with inflammatory pain investigating the two pathways, p38MAPK and ERK. Junying Du et al. [13] discussed the possibility of the involvement of p38MAPK pathway in inflammatory pain and electroacupuncture intervention and hypothesized that the initiation of p38MAPK signaling plays an important role in the production and maintenance of inflammatory hyperalgesia and can augment intervention with electroacupuncture. This study group [14] further explored the potential correlation and found that electroacupuncture could reduce the protein expression of p-p38MAPK and p-ATF-2, reduce the level of p-p38 MAPK-IR and p-ATF-2-IR in the spinal dorsal horn cells of CFA rats, and inhibit the expression of VR-1 protein and gene. It was suggested that inhibition of the activation of the p38 MAPK/ATF-2/VR-1 pathway in the spinal cord may be one of the main mechanisms of the central signal transduction pathway responsible for anti-inflammatory processes in CFA rats. The research group of Huazhong Agricultural University revealed the mechanism of anti-inflammation and analgesia effects of electroacupuncture and explained that electroacupuncture can inhibit both the PAG-RVM-SCDH p38MAPK level in the central nervous descending pathway and the PAG-ACB-AMY-Hb p-p38MAPK level, thereby reducing the level of hyperalgesia in rats with inflammatory pain. Du K et al. [15] stimulated “Kunlun point” (BL60), “Zusanli point” (ST36), and “Sanyinjiao point” (SP6) in inflammatory pain model in rats and demonstrated painless hypersensitivity effects by regulating the expression of p38 MAPK. Ji et al. [16] found that the activation of ERK resulted in an increased expression of prodynorphin mRNA and substance P receptor neurokinin (NK-1) and thus augmented hyperalgesia in adjuvant arthritis rats to thermal and mechanical injury stimuli. Chunlei Wang et al. [11] showed that increased phosphorylation of ERK also played an important role in the development of pain and analgesia effects of electroacupuncturing Jiaji point (Ex-B2). Electroacupuncture may exert its analgesic effects by regulating the expression of phosphorylated ERK (p-ERK) in the spinal dorsal horn of adjuvant arthritis rats. In addition, more studies have also proven the effects of electroacupuncture on inflammatory pain. Zhou jie et al. [17] confirmed that the combination of different parameters of electroacupuncture can alleviate inflammatory pain in rats. The combination of different parameters of electroacupuncture has the same analgesic effects in the early stage of inflammatory pain, while electroacupuncture of 2/100hz or 2/120hz frequency has better effects in the later stage. Lv ZT et al. [18] showed that electroacupuncture can relieve the pain of knee osteoarthritis, and strong electroacupuncture stimulation is better than weak electroacupuncture stimulation. However, the authors did not further explain the mechanism of electroacupuncture mitigating inflammatory pain.

### 3.2. Antineuropathic Pain Effects of Electroacupuncture

One form of expression of neural plasticity is neuropathic pain. Persistent noxious stimuli produced by nerve injury lead to central sensitization, which is an activity-dependent functional plasticity change. MAPK cascade-associated signaling pathway is considered as the main valve for the regulation of multiple signal pathways in cells during central sensitization of pain. Huan Yang et al. [19] used electroacupuncture to stimulate the pain threshold in rats with diabetic neuropathic pain and showed that electroacupuncture can not only increase the pain threshold of DNP rats but also reduce blood glucose level. The mechanism may be related to the inhibition of the expression of p38MAPK in the spinal cord. The mechanism of this conclusion is similar to that of the study by Zhenhua Cai et al. [20]. They also selected the electroacupuncture Zusanli point (ST36) to inhibit the expression of p38MAPK in the dorsal root ganglion of rats. The purpose of alleviating thermal pain and mechanical pain in rats with neuropathic pain and improving motor function score of affected hind limbs was achieved. For chronic neuropathic pain, multiple electroacupuncture can increase the activity of Ras/Raf/ERK signaling pathway and the expression of p38MAPK, which indicates that the cumulative effect of electroacupuncture may be associated with the changes in the Ras/Raf/ERK signaling pathway and the expression of p38MAPK to achieve the analgesic effects [21]. Deng G [22] and Jiang S [23] have revealed the role of electroacupuncture in neuroprotection and neuropathic pain in specific acupoints, and studies by Deng G et al. have shown that amelioration of pain by electric acupuncture is associated with improved anxiety and mood disorders simultaneously, thereby promoting recovery. However, the study of electroacupuncture against neuropathic pain does not involve the MAPK signaling pathway, and whether it is correlated with the MAPK signaling pathway remains to be evaluated by further study.

### 3.3. Analgesic Effects of Electroacupuncture against Labor Pain

Labor pain includes visceral pain (visceral nervous system transmitted to the spinal nerves) and somatic pain (compression of the pelvic nerve fibers), which by pain index ranks second only to pain from burn injury [24]. There are no reports demonstrating that electroacupuncture may have side effects during labor analgesia. Although studies have found that *β*-endorphin secretion may be a causative factor in the analgesic effects of electroacupuncture against labor pain, the mechanism at this stage is not yet clear [25]. In China, Meili Wang and Yingying Qin expounded the effects of electroacupuncture on the expression of the downstream signal molecules in the spinal cord from two signal pathways, MAPK/ERK, and MAPK/p38. Jiang Qiuyan et al. [26] studied 320 women and clarified that electroacupuncture can reduce the expression of EP2 by inhibiting the activation of p38 and its downstream signaling molecules, thereby reducing labor pain. Wang Mengying [27] found that music and electroacupuncture have a positive effect on reducing labor pain. The mechanism may be through inhibition of MAPK/ERK signaling molecules, C-Raf and ERK, CREB mRNA levels, and phosphorylation of ERK and CREB protein. Increasing the expression of DYN effector molecule mRNA and protein in the placental tissue and its concentration in the serum could finally achieve analgesic effects. Therefore, the mechanism of electroacupuncture in relieving labor pain needs further study to lay a solid foundation for clinical application.

## 4. Mechanism of Protection by Electroacupuncture against Damage

Studies on the role of MAPK cascade-related signal pathway on the protective effects of electroacupuncture are still limited. The protective mechanisms of acute lung injury and posttraumatic immunosuppression have been less explored in China and overseas. The lung is the most common organ to be injured by endotoxic shock. The syndrome can escalate to acute respiratory distress syndrome in a short period of time and even multiple organ failure syndrome which eventually leads to death. Scholars from the Tianjin Medical University found that PKCa and p38MAPK pathways are involved in the electroacupuncture-mediated upregulation of HO-1 expression during acute lung injury in rabbits with endotoxic shock [28]. The protective role of Keap 1-Nrf2/ARE pathway in electroacupuncture-relieved lung injury induced by endotoxic shock in rabbits is related to its phosphorylation, which promoted nuclear translocation of Nrf2 protein by p38MAPK. This means that p38MAPK signaling played a crucial role in protection against lung injury [29]. Chinese scholars [30] further established a lung injury model in rats to study the protective mechanism of electroacupuncture in acute lung injury induced by cardiopulmonary bypass and found that electroacupuncture can reverse pulmonary inflammation, oxidative damage, and apoptosis. This mechanism might be directly related to the caspase-3 activity and the reduction of p38 phosphorylation. However, Jing Li creatively proposed that by electroacupuncturing the Zusanli Point (ST36), phosphorylation of ERKS and ERK1/2 signaling pathways in the cerebral cortex gets inhibited, and thereby, the immunosuppression caused by traumatic stress can be improved. This filled the gap in the research on the protective mechanism of electroacupuncture injury in China and abroad.

## 5. The Role of Electroacupuncture in Regulating Gastrointestinal Functions

Many studies have confirmed that acupuncture in the stomach meridian point, especially in the Zusanli point (ST36), has a benign regulatory effect on the digestive system, especially on the gastrointestinal functions. Although many studies have shown that neuroimmune-endocrine networks, nerve nuclei, neurotransmitters, body fluids, and gastrointestinal hormones are all involved in the beneficial effects of acupuncture in regulating gastrointestinal functions, the mechanism of electroacupuncture regulating the gastrointestinal functions is rarely seen from the perspective of MAPK signal transduction regulation. Qi Yang et al. [31] found that the frequency and amplitude of electrogastrogram were increased when rats were electroacupunctured on the Zusanli point (ST36). Finally, it was suggested that the mechanism of electroacupuncture to promote gastric movement might be mediated through the ERK pathway by the ICCs and MAPK signal transduction. No further research on the mechanism was conducted since then. Zongbao Yang et al. [32] observed that serum extracted from rats with an electroacupuncture gastric mucosal injury can significantly upregulate ERK phosphorylation in the gastric mucosal cells, which was significantly different from that in the normal and model rats. After electroacupuncture on the Weishu point (BL21), the effect of serum was significantly stronger than that in serum after electroacupuncture on the Dannang point (EX-LE6), suggesting that ERK might be involved in the signal transduction of electroacupuncture on the Weishu point (BL21) during the repair of gastric mucosal injury. In recent years, Q Yang et al. [33] have elaborated this mechanism of action by suggesting that electroacupuncturing the rats on the Zusanli point (ST36) may double the effects based on different frequency intensities. Gastric exercise elevation may be related to the upregulation of MAPK6 (ERK3) and downregulation of IL1R2, whereas gastric exercise inhibition may be associated with the upregulation of FOS, CXCL9 (MIG), and NOS2A (iNOS) and downregulation of MMP9. In summary, the PKC and MAPK pathways may be two related signaling pathways involved in the regulation of gastrointestinal functions by electroacupuncture.

## 6. Effects of Electroacupuncture on Cardiovascular and Cerebrovascular Diseases

In recent years, research on electrocardio therapy for cardiovascular and cerebrovascular diseases and exploration of the underlying mechanism at home and abroad has been deepened and diversified. For cerebrovascular diseases, it mainly involves cerebral ischemia-reperfusion injury, Alzheimer's disease, cardiomyopathy, Parkinson's disease, vascular dementia, hypertension, etc. For cardiovascular disease, the mechanisms of myocardial ischemia-reperfusion and cardiac hypertrophy are mainly studied.

### 6.1. Intervention on Cerebral Ischemia-Reperfusion Injury

The incidence of cerebral ischemia-reperfusion injury is on the rise, accounting for about 80% of all types of cerebrovascular disease [34]. Cerebral cell necrosis and apoptosis are induced during cerebral ischemia and hypoxia, and the body also initiates a self-repairing mechanism to protect the brain neurons. Current research shows that acupuncture has a definite curative effect on the treatment of cerebral ischemia-reperfusion injury, and it plays an important role in improving microcirculation, improving brain electrical activity, and inhibiting oxygen-free radicals, thereby greatly reducing nerve injury [35, 36]. The signal transduction pathway involved in cerebral ischemia repair is mainly the MAPK cascade transduction pathway, which includes three pathways, such as ERK, JNK, and p38. Research by Dengjun Guo et al. [37] showed that acupuncture can promote the recovery of neurological functions in rats with ischemic brain injury and reduce the volume of cerebral infarction. It is speculated that the mechanism of acupuncture may involve benign regulation of the reactivity of the MAPK signaling pathway through inhibition. The activity of the MAPK pathway reduced ERK-1/2 phosphorylation and upregulated the expression of glial neurotrophic factor (GDNF) in the brain tissue around the ischemic focus. The research of Meihong Shen et al. [38] showed that electroacupuncture can upregulate ERK protein expression in injured neurons of rats with cerebral ischemia-reperfusion. It is speculated that this is the mechanism by which electroacupuncture mitigate oxidative stress and can initiate cytothesis, and thus, promote cell survival. Zhonghua Yang et al. [39] found that electroacupuncture can regulate the MAPK/ERK signaling pathway, reduce the expression of p-ERK in the early stage of cerebral ischemia, and finally interfere with the potential toxic effects on neurons thereby protecting the brain. Based on previous research, Lan X et al. [40] observed the effects of electroacupuncture stimulation on two groups of acupoints “Chize point (LU5), Hegu point (LI4)” and “Zusanli point (ST36), Sanyinjiao point (SP6)” on the neurological deficit score, brain tissue morphology, apoptosis, and phosphorylated p38MAPK in rats with cerebral ischemia-reperfusion injury. “Electroacupuncture stimulation-initiated p38MAPK signaling regulation-antagonize apoptosis” was found to be one of the possible mechanisms of electroacupuncture treatment of the disease. The authors also observed that the third day might be a better time to repair the cerebral ischemic nerve. The p38 and ERK of the MAPK family signaling pathways are in the focus of research in recent years. Under the effects of electroacupuncture, it can reduce nerve injury after cerebral ischemia and reperfusion and can provide a new target for the prevention and treatment of cerebrovascular diseases.

### 6.2. Intervention in Alzheimer's Disease

Patients with Alzheimer's disease generally have a strong focal inflammatory reaction in the brain. Many activated glial cells and inflammatory reactive substances can be generated around the senile plaque. The p38MAPK signaling pathway plays an important role in the inflammatory response. Jianqiao Fang et al. [41] selected the electroacupuncture Baihui point (GV20), Taixi point (KI3), and Zusanli point (ST36), each for 15 min, once per day, 6 times as a single course, and restarted one course after a day's break. Electroporation was found to regulate the phosphorylation of p38MAPK in Alzheimer's disease model rats, thereby blocking its inflammatory response. Meanwhile, it is proposed that the regulation of electroacupuncture itself is still limited. It is also necessary to expand the sample and increase the treatment course for further exploration. Min Zhang et al. [42] selected Baihui point (GV20) and bilateral Shenshu point (BL23) for electroacupuncture. Using Morris water maze, memory capability, immunohistochemistry, and the expression of p-p38MAPK and p-tau Thr181 protein in the hippocampus of the rats were detected. The results showed that electroacupuncture can reduce the expression of p-tau protein in the hippocampus of rats with Alzheimer's disease by regulating the p38MAPK pathway and can improve learning and memory ability. All these findings are of great contribution to exploring the mechanism of electroacupuncture in the prevention and treatment of Alzheimer's disease.

### 6.3. Intervention of Myocardial-Related Diseases

The number of people who die of acute myocardial infarction every year in China has exceeded one million, and the incidence and the mortality rate are still rising. The mechanism of myocardial ischemia-reperfusion injury includes many aspects, such as oxidative stress, inflammatory response, mitochondrial injury, calcium overload, energy imbalance, and apoptosis [43, 44]. p38MAPK through a three-stage enzymatic cascade for signal transduction can be activated by a variety of stress stimuli, such as hypertonic, ischemia-reperfusion, etc. Phosphorylated p38MAPK can further activate the downstream substrates, activate inflammatory cells, and release inflammatory cytokines, such as tumor necrosis factor and interleukin until apoptosis [45]. Its role in improving cardiomyocyte activity during myocardial ischemia-reperfusion injury is crucial. Song Chen et al. [46] used electroacupuncture “Neiguan point (PC6)” pretreatment to improve cardiomyocyte activity, reduce myocardial infarct size, and protect mitochondrial integrity. It can significantly inhibit the expression of MKK3/6 and p38MAPK in ischemia-reperfusion injury and inhibit the phosphorylation of p38MAPK, suggesting that this may be one of the mechanisms of electroacupuncture intervention in ischemia-reperfusion injury of the myocardium. Chinese scholars continue to use electroacupuncture on the Neiguan point (PC6) to further study myocardial ischemia-reperfusion injury. Starting from the characteristics of the whole gene expression, it was found that 725 genes were upregulated, and 861 genes were downregulated, and some of the gene expression could be reversed. It also reveals that electroacupuncture can effectively protect the cardiomyocytes, and the mechanism is closely related to the regulation of the MAPK signaling pathway in the damaged myocardial tissues [47]. For the “Neiguan point (PC6)”, by using continuous wave frequency of 2 Hz, intensity of 1 mA, and 14 days of electrification for 20 min, the phosphorylation of p38MAPK was significantly inhibited in the hypertrophic rats. The mechanism of action may be through the inhibition of the expression of inflammatory factors, such as TNF-*α*, and IL-1*β* which eventually regulate the p38MAPK signaling pathway [48].

### 6.4. The Role of Regulating Hypertension

A study [49] showed that the proliferation of vascular smooth muscle cells is responsible for causing the main lesion of hypertension, and its occurrence is closely related to the overactivation of ERK in MAPK. Mitogen-activated protein kinase phosphatase 1 (MKP-1), an important factor in the dephosphorylation of the MAPK signal transduction pathway, plays an important role in this pathway. Lin Jiang et al. [50] through electroacupuncture of bilateral “Fhengchi point (GB20)”, “Quchi point (LI11)”, and “Sanyinjiao point (SP6)” observed that the mRNA protein expression of MKP-1 in the aorta of spontaneously hypertensive rats was upregulated, and the level of phosphorylation of ERK-1/2 protein was downregulated, indicating that acupuncture treatment could effectively improve the expression of ERK protein activity in the body, reduce MAPK activity, and inhibit the proliferation/hypertrophy of vascular smooth muscle cells. It is presumed that this may be an important molecular mechanism for acupuncture and moxibustion to regulate blood pressure and improve cardiovascular remodeling.

### 6.5. Intervention in Other Brain Diseases

The effect and mechanism of electroacupuncture on Parkinson's disease and vascular dementia have been discussed by some other scholars. However, the molecular mechanism involved in the MAPK family signaling pathway remained unclear. Studies have shown that electroacupuncture can reduce the expression of p-ERK 1/2 and p-c-Jun in the substantia nigra by decreasing the levels of MAPK/ERK 1/2 and MAPK/JNK in a rat model of Parkinson's disease. The reduced TNF-*α* and IL-1*β* levels and other protein expression play a certain role in the development of Parkinson's disease [51, 52]. In vascular dementia, the regulation of the p38MAPK pathway is still of interest to the scholars. Neurons in the hippocampus repaired after electroacupuncture demonstrated cognitive improvement after vascular dementia. A 2/100Hz frequency electroacupuncture stimulation of “Dazhui point (BL11)”, “Baihui point (GV20)”, “Zusanli point (ST36)”, and “Geshu point (BL17)” can improve cognitive impairment in rats. The mechanism may affect apoptosis of hippocampal neurons in rats, which is mediated by p38MAPK, Bcl-2, Bax, and Caspase-3. Electroacupuncture points can inhibit the expression of p38MAPK, suggesting that it may be through inhibition of p38MAPK signaling pathway. Both reduce the secondary nerve injury and eventually promote the repair of damaged neurons during the intervention.

### 6.6. Intervention in Other Diseases

Electroacupuncture treatment of skin, knee joints, and other diseases based on MAPK family signaling pathways have been rarely reported domestic and overseas. Libo Chou et al. preliminary found that the mechanism of electroacupuncture in promoting healing of skin lesions in pressure sore rats may be related to the regulation of p38 MAPK mRNA and protein expression levels in rat skin tissue [53]. Z Wang et al. [54] first proposed that electroacupuncture at Zusanli point (ST36) can improve allergic contact dermatitis-related inflammation in rats by regulating IL-10 production and inhibiting p38 MAPK activation. This provides an alternative and promising treatment method for allergic contact dermatitis. In the current study of knee articular chondrocytes, there are two feasible molecular mechanisms. First, electroacupuncture inhibits the expression of apoptotic genes p38 and Fas mRNA and reduces the expression of MAPK signaling pathway. Second, by reducing the phosphorylation of ASIC1 protein and p38MAPK in chondrocytes, the expression of apoptotic factor p53 is inhibited, and therefore, results in the inhibition of apoptosis in the chondrocytes [55, 56].

## 7. Conclusion and Outlook

MAPK is a type of serine/threonine protein kinase widely found in mammals and can be activated by a series of extracellular signals or stimuli, such as physical stress, inflammatory cytokines, growth factors, and bacterial complexes. MAPKs are components of a series of cascade reactions that are key factors of a variety of extracellular stimuli and can regulate basic cellular processes. MAPK signal transduction is carried out in the form of a cascade of three kinases. First, MAPKKK is activated by mitogen-induced phosphorylation, then MAPKKK phosphorylates and activates MAPKK, and finally, MAPK is phosphorylated and activated by MAPKK, and then transferred into the nucleus [57, 58].

Since 1991, Sturgill et al. identified extracellular signal-regulated kinase (ERK) in the mammalian cells, and research on MAPK signaling pathway has developed rapidly since then [59]. Till now, eight MAPK subfamilies have been identified, namely, 38MAPK, ERK-1/2, c-jun N-terminal kinase (JNK), ERK-3, large mitogen-activated protein kinase-1 (ERKS/BMK1), ERK-7, Nemo-like kinase (NLK), and ERK-8. These subfamilies constitute multiple pathways, among which are the ERK-1/2 pathway, the JNK pathway, and the p38 pathway [60]. MAPK function has the characteristic of cell type and condition dependency. The biological responses caused by various MAPK pathways are different: ERK pathway mainly regulates cell growth and survival and can also promote cell differentiation under certain conditions. JNK and p38 pathways are mostly activated under stress conditions and mainly mediate proapoptotic signals, growth inhibitory signals, and inflammatory responses. Under certain conditions, the p38 pathway can also induce antiapoptotic, proliferative, and cell survival signals, depending on the tissues and subtypes of p38 involved.

Electroacupuncture stimulation therapy is a treatment method in which electrostimulation is applied based on acupuncture to enhance the treatment effect. It has double benefits of acupuncture and electric stimulation. In the past, the bidirectional regulatory mechanism of electroacupuncture over the whole body was mostly involved in the neurobody fluid level. At present, from the molecular biology aspect, the study of electroacupuncture therapy based on signal transduction has become a hot topic related to the regulation of overall physiology and pathology.

The most recent studies have demonstrated the role of electroacupuncture in the treatment of MAPK signal transduction pathways and its mechanisms have been effectively verified and reliably stated. (1) In terms of electroacupuncture analgesia, the anti-inflammatory and analgesic mechanisms of electroacupuncture may include the following: analgesic effect was achieved by inhibiting of the activation of the p38 MAPK/ATF-2/VR-1 pathway, inhibiting both the PAG-RVM-SCDH p38MAPK level in the central nervous descending pathway and the PAG-ACB-AMY-Hb p-p38MAPK level and regulating the expression of phosphorylated ERK (p-ERK) in the spinal dorsal horn. And the mechanism of electroacupuncture may inhibit the expression of Ras/Raf/ERK signaling pathway and p38MAPK to achieve the analgesic effects. In addition, electroacupuncture may reduce MAPK/ERK and MAPK/p38 signaling pathways to achieve the effect of antilabor pain. (2) In the treatment of lung injury by electroacupuncture, the following mechanisms may play key roles: (1) It may be related to the upregulation of HO-1 expression mediated by p38MAPK pathway, and p38MAPK plays a key role in the protection of lung injury in the Keap 1-Nrf2/ARE pathway. (2) It is possible to improve the immunosuppression caused by traumatic stress by inhibiting the phosphorylation of ERKS and ERK1/2 signaling pathways in the cerebral cortex. (3) In terms of the regulation of gastrointestinal function by electroacupuncture, PKC and MAPK pathway may be the two related signaling pathways in its therapeutic mechanism, and gastric exercise elevation may be related to the upregulation of MAPK6 (ERK3) and downregulation of IL1R2, whereas gastric exercise inhibition may be associated with the upregulation of FOS, CXCL9 (MIG), and NOS2A (iNOS) and downregulation of MMP9. (4) In the treatment of cardiovascular and cerebrovascular diseases, as for the intervention of electroacupuncture on ischemia-reperfusion injury, the three pathways involved in the MAPK cascade transduction pathway, namely, ERK, JNK, and p38, are mainly involved. Among them, the p38 and ERK of the MAPK family signaling pathways are in the focus of research in recent years, which can provide a new target for the prevention and treatment of cerebrovascular diseases. The mechanism of AD treatment may be related to the regulation of p38MAPK pathway and the reduction of p-tau protein expression in the hippocampus of rats. In addition, p38MAPK pathway also plays an important role in the improvement of myocardial ischemia mechanism by electroacupuncture. It may inhibit the phosphorylation of p38MAPK by inhibiting the expression of MKK3/6, p38MAPK protein in l/R and TNF-*α*, IL-1*β*, and other inflammatory factors, thus achieving therapeutic effect. Furthermore, the improvement of hypertension by electroacupuncture may be related to the improvement of expression of ERK protein activity in the body and the reduction of MAPK activity, thereby participating in the inhibition of proliferation/hypertrophy of vascular smooth muscle cells. Finally, a few numbers of studies have suggested that the p38MAPK pathway may also be one of the mechanisms to improve Parkinson's disease and vascular dementia. (5) In the interventions for other diseases, reports electroacupuncture in the treatment of other diseases based on the MAPK family signaling pathway have rarely been reported, only in the treatment of skin and knee diseases involved, and not perfect and in-depth.

All the above research conclusions are based on the level of animal experimental research. Meanwhile, clinical researches on electroacupuncture based on the MAPK signaling pathway mechanism are also being reported, realizing the transformation from basic experimental research to clinical application, especially in the field of anti-inflammatory and analgesic, such as the effective verification in the field of antilabor pain. However, electroacupuncture has different prescription treatment for each disease in clinical treatment, and its efficacy is closely related to the frequency, waveform, treatment course and acupoint selection et al. Therefore, based on the theoretical basis of these molecular mechanisms, we could observe the changes in the efficacy and molecular mechanism of electroacupuncture through the selection of different electroacupuncture frequency, waveform, course of treatment and acupoints, and then select the optimal therapy of various diseases, which would provide a solid theoretical basis for the treatment of frequently occurring and common diseases with electroacupuncture.

However, there are still some problems that need to be solved by current researches. (1) Most of the studies need to be more in-depth and more cross-sectional. The relationship between the electroacupuncture effect and regulation of MAPK family signal transduction pathways needs further research from various perspectives. (2) Although the current researches involve three pathways of p38MAPK, ERK-1/2, and c-JunJNK, they are focused on changes of p38MAPK-related proteins and genes and lack systematic studies on the changes of p38MAPK downstream factors. Researches are also limited to the observation of members of a single MAPK signaling pathway, and there is a lack of research on the functions of multitargeted, deep-level interactions. (3) Almost all the above results are based on animal model experiments and there is a lack of clinical regression and validation. There is no systematic review r analysis, and there is a lack of multicenter, multigroup, large sample, stratified control group, and other clinical research methods. Therefore, the true long-term efficacy and comparison remain to be explored. (4) There is a lack of variant including electroacupuncture waveform, frequency, stimulation intensity, acupoint selection points, etc.

In summary, the electroacupuncture intervention of the MAPK signaling pathway can effectively treat a variety of diseases. However, the current research results are mostly supported by experimental animal evidence, and there is a lack of clinical data. It is believed that with the improvement in medical technology and continuous updation of research techniques, such as proteomics, metabolomics, and apoptosis, further data will be generated. The mechanism of electroacupuncture-induced alteration of the MAPK signaling pathway will be elucidated in more detail to clarify its importance to the improvement of clinical efficacy of electroacupuncture.

## Figures and Tables

**Table 1:** List of electroacupuncture treatment-related diseases based on MAPK signal pathway.

Effect	MAPK signal pathway	Number of articles	Summary
Analgesia	p38MAPK, ERK	19	The mechanism of anti-inflammatory effects and amelioration of neuropathic pain is more complete, while that of the curative effect is specific. There are few reports on anti-delivery pain, and the specific mechanism needs to be further clarified.

Resisting injury accelerates injury repair	p38MAPK, ERK	3	Few studies have reported advantages in acute lung injury and post-traumatic immunosuppression.

Regulate gastrointestinal functions	ERK	3	Few studies have reported the advantages of gastric mucosa repair and maintenance of gastrointestinal functions.

Intervention in cardiovascular and cerebrovascular diseases	p38MAPK, ERK, JNK	19	It has effective regulatory effects on cerebral ischemia-reperfusion, Alzheimer's disease, cardiomyopathy, Parkinson's disease, and hypertension.

Other effects	p38MAPK	3	Few studies have reported the advantages of electroacupuncture in allergic dermatitis and knee diseases.
